# Depression and Incidence of Frailty in Older People From Six Latin American Countries

**DOI:** 10.1016/j.jagp.2019.04.008

**Published:** 2019-10

**Authors:** A. Matthew Prina, Brendon Stubbs, Nicola Veronese, Mariella Guerra, Carolina Kralj, Juan J. Llibre Rodriguez, Martin Prince, Yu-Tzu Wu

**Affiliations:** 1 Social Epidemiology Research Group (AMP, Y-TW), Deparment of Health Service and Population Research, Institute of Psychiatry, Psychology & Neuroscience, King's College London, London; 2 Global Health Institute (AMP, MP), King's College London, London; 3 Department of Psychological Medicine (BS), Institute of Psychiatry, Psychology & Neuroscience, London; 4 South London and Maudsley NHS Foundation Trust (BS, CK), London; 5 National Research Council, Neuroscience Institute (NV), Aging Branch, Padua, Italy; 6 Ambulatory of Nutrition (NV), IRCCS “S. de Bellis” National Institute of Gastroenterology–Research Hospital, Castellana Grotte, Bari, Italy; 7 Institute of Memory, Depression and Disease Risk (MG), Lima, Peru; 8 Facultad de Medicina Finlay-Albarran (JJLR), Medical University of Havana, Havana, Cuba

**Keywords:** Depression, older age, epidemiology, frailty, low- and middle-income countries

## Abstract

**Objective:**

Frailty and depression are highly comorbid conditions, but the casual direction is unclear and has not been explored in low- and middle-income countries. The aim of this study was to investigate the potential impact of depression on incident frailty in older people living in Latin America.

**Methods:**

This study was based on a population-based cohort of 12,844 people aged 65 or older from six Latin American countries (Cuba, Dominican Republic, Mexico, Venezuela, Puerto Rico, and Peru), part of the 10/66 cohort study. Two types of frailty measures were used: a modified Fried frailty phenotype and a multidimensional frailty criterion, which included measures from cognition, sensory, nutrition, and physical dimensions. Depression was assessed using EURO-D and *International Classification of Diseases, Tenth Revision* criteria. A competing risk model was used to examine the associations between baseline depression and incidence of frailty in the 3–5 years of follow-up, accounting for sociodemographic and health factors and the competing event of frailty-free death.

**Results:**

Depression was associated with a 59% increased hazard of developing frailty using the modified Fried phenotype (subdistribution hazard ratio [SHR]: 1.59; 95% confidence interval [CI]: 1.40, 1.80) and 19% for multidimensional frailty (SHR: 1.19; 95% CI: 1.06, 1.33) after adjusting for sociodemographic factors, physical impairments, and dementia. The associations between depression and the multidimensional frailty criteria were homogenous across all the sites (Higgins I^2^ = 0%).

**Conclusion:**

Depression may play a key role in the development of frailty. Pathways addressing the association between physical and mental health in older people need to be further investigated in future research.

## Highlights

• **What is the primary question addressed by this study?**
  What is the association between depression and incidence of frailty in older people from six Latin American countries?
• **What is the main finding of this study?**
  Depression was associated with an increased risk of developing frailty, either the modified Fried phenotype or multi-dimensional frailty. The strength of associations for multi-dimensional frailty were homogenous across six countries.
• **What is the meaning of the finding?**
  Depression may play a key role in the development of frailty and underlying mechanisms need to be investigated in future research.

## INTRODUCTION

Frailty is an age-related biological syndrome resulting in decreased physiological reserve and increased susceptibility to stressors during the aging process,^1^, ^2^ and ultimately in increased disability and mortality.^3^, ^4^ A meta-analysis estimated the prevalence of frailty to be 11% in community-dwelling older people, but range was wide across studies (4%–59%).^5^ A multicenter cohort study in five Latin American cities reported relatively high estimates in both men (21%–35%) and women (30%–48%).^6^

Similar to frailty, depression is also a highly prevalent condition among older adults and has been linked to an increased risk of developing frailty in later life.^7^ Although recent reviews have suggested bidirectional associations between depression and frailty in later life,^8^, ^9^, ^10^, ^11^ it is important to investigate how depression might lead to incident frailty particularly in low- and middle-income countries, where high prevalence of depression has been reported in some settings but access to health services are limited.^12^ Given the large number of older people in low- and middle-income countries, population-based longitudinal studies are needed to quantify the potential impact of depression on the development of frailty. This may lead to the identification of a high-risk group of individuals who are likely to become frail, potentially leading to a reduction in burden associated with both depression and frailty.

Based on the pooled estimates of four longitudinal studies from the United States and Germany, older people with depression had four-fold increased odds of incident frailty, yet the reported heterogeneity of this meta-analysis was high.^8^ This might be related to variation in research methods such as different measures for depression and frailty. In addition to the classic Fried criteria, which focuses on five clinical markers related to declines in physical functioning,^13^ the multidimensional nature of frailty has been widely recognized in recent years,^14^ and several assessment methods have been developed to incorporate different dimensions of physical and mental health indicators.^2,^^13^, ^14^, ^15^, ^16^ However, few studies have included different frailty definitions and examined their effects on the association between depression and frailty. If the association varied across different frailty definitions, this might clarify possible pathways between depression and frailty.

Using a population-based cohort of older people living in six Latin American countries, the aim of this study was to investigate the potential association between depression and incident frailty in later life. Moreover, we explored whether the associations maintain when a different definition of frailty is considered.

## METHODS

### Sample

The 10/66 Dementia Research Group carried out surveys of older people aged 65 and over living in 11 catchment areas across eight low- and middle-income countries (China, Cuba, Dominican Republic, India, Mexico, Peru, Puerto Rico, and Venezuela). One urban and one rural site were present in China, Mexico, and Peru, whereas the other countries only included an urban site. The catchment area boundaries were well-defined, and areas with high-income earners were avoided. The baseline surveys took place between 2003 and 2005 for all sites, with the exception of Puerto Rico, in which data were collected from 2007. A full follow-up was carried out 3–5 years after the baseline, and date of death of those deceased were also recorded. Informed consent was obtained from all participants and verbal consent was used when participants were illiterate. The study was approved by local ethical committees and by the King's College London research ethics committee. Full details of the protocol and the cohort are available elsewhere.^17^, ^18^

This study only focused on a subset of the full 10/66 dataset (N = 15,901), using 12,844 participants from the six Latin American countries. The Indian sites were excluded due to incomplete follow-up data. Compared with Latin American countries, the prevalence of depression was found to be markedly low in the Chinese sites^12^ and there was lack of statistical power to investigate its association with incident frailty.

### Measurement

Two types of frailty definitions were used in this analysis. The original Fried frailty phenotype includes five indicators: exhaustion, weight loss, weak grip strength, slow walking speed, and low energy expenditure. The 10/66 cohort study assessed only four of the five indicators and did not include measures of hand grip strength.^19^, ^20^ Self-reported measures of exhaustion, weight loss (≥10 lb in the last 3 months), and low energy expenditure (physically inactive) were included in the interviews. Walking speed was assessed using a time walking test (5 meters at usual speed, turn and return to the starting point) and the slowest quintile by sex and height stratum in each catchment area was considered to have a slow walking speed. Participants were defined as frail if they had two or more of the four frailty indicators, as done in previous studies.^19^, ^20^ To align with the literature,^2^ a cutoff of three or four frailty indicators was also applied, yet very few people belonged to this category (Supplementary Table S1).

The multidimensional frailty approach was developed in the Alameda County study^21^ and previously used by our group.^19^, ^20^ It includes 16 self-reported items that form four broad domains of functioning (cognitive, nutrition, physical, and sensory). The cognitive functioning domain included attention difficulties and memory. The nutrition domain included unexplained weight loss and loss of appetite. The physical functioning domain included items measuring balance loss, dizziness, and weakness in limbs. Finally, the sensory functioning domain included hearing and vision difficulties. If difficulties in two or more domains were present, participants were considered frail.

Considering that *International Classification of Diseases, Tenth Revision* (ICD-10) criteria were not specifically developed for older adults and might under-detect depression in later life, depression in this study was determined using both ICD-10 criteria, which was generated using specific Geriatric Mental State Examination (GMS) algorithms,^12^, ^22^ and the EURO-D scale.^23^, ^24^ The EURO-D scale, which was developed to compare symptoms of late-life depression across 11 European countries, has 12 items including depressed mood, pessimism, wishing death, guilt, sleep, interest, irritability, appetite, fatigue, concentration, enjoyment, and tearfulness. Each item has a score of 0 (symptom not present) or 1 (symptom present), with a total score range between 0 and 12. Participants who met the ICD-10 depression criteria or had a EURO-D score of 4 or 5 were considered to have depression. Procedures to select the optimal cutoff of 4 or 5 on the EURO-D scale have been reported in the EURO-D validation articles, showing high sensitivity and specificity to a diagnosis of clinical depression in low- and middle-income countries.^23^, ^24^

Sociodemographic characteristics including age, sex, and education (none/did not complete primary, completed primary, secondary, tertiary) were collected in the interviews. The measure for limiting physical impairments was based on 12 items of common physical impairments,^25^ including arthritis/rheumatism, eyesight problems, hearing difficulty or deafness, persistent cough, breathlessness/asthma, high blood pressure, heart trouble/angina, stomach problems, intestine problems, faints/blackouts, skin disorders, and paralysis/weakness or loss of one leg or an arm. Impairments were rated as present if they interfered with activities “a little” or “a lot,” as opposed to “not at all.” The total number was then categorized into three groups: none, one or two, or three or more. Dementia was assessed using the 10/66 dementia diagnosis adjusted for education, which has been widely used in previous articles from the 10/66 Dementia Research Group, showing strong psychometric properties. Further information on this measure is available elsewhere.^26^

### Statistical Analysis

Before regression modeling, we reported the percentage of incident frailty by baseline depression status excluding those participants with frailty, either the modified Fried phenotype (N = 2,375) or multidimensional frailty (N = 3,886) at baseline. Because mortality was considered to be a competing outcome to frailty in later life, a competing risk model was used to investigate the associations between depression and incidence of frailty. Subhazard ratio estimates have similar interpretation to hazard ratios but also account for a competing event (frailty-free mortality). The proportional subhazard assumption was assessed generating time dependent covariates, by adding interactions of the predictors and a function of survival time in the assessed model. Two types of frailty outcomes, the modified Fried phenotype and multidimensional frailty, were modeled separately. Adjusted models included sociodemographic factors (age, sex, and education), number of limiting physical impairment, and dementia, which is related to depression and incident frailty. The unadjusted and adjusted models were conducted for each country and pooled estimates of all six countries were generated using a fixed effect meta-analysis. Higgins I^2^, an indicator for the level of heterogeneity,^27^ was used to assess variation in effect sizes across the six countries.

Inverse probability weighting was used to examine the potential impact of participants lost to follow-up (N = 1,369; 13.1%) on the results. Weights were generated using all variables in the fully adjusted model and country and were applied to all competing risk models. Because the weighted estimates were similar to the unweighted ones, the results of complete case analysis are reported here. A sensitivity analysis was carried out to exclude people with dementia at baseline and examine whether the associations were different in the participants without dementia. All analyses were conducted using Stata version 15.1 (StataCorp LLC, College Station, TX).

## RESULTS

Descriptive information on the baseline study population is provided in Table 1. Among the 12,844 participants, the mean age was 74.7 (standard deviation: 7.2) years and 64.5% were women. Nearly 40% had none or some education but did not complete primary school. The proportion of people with dementia was 10%, and 17.8% had three or more limiting physical impairments. For frailty, 18.5% of participants had modified Fried phenotype (two or more characteristics), and 30.3% had multidimensional frailty. More detailed information on the numbers of Fried frailty characteristics is reported in Supplementary Table S1. Approximately 12% (N = 1,546) of participants were identified as having frailty using both definitions. The proportion of people with depression at baseline was 26.9% with a range between 16.5% in Puerto Rico and 37.9% in Dominican Republic.

**TABLE 1:** Descriptive Information on the Study Population at Baseline

	Cuba	Dominican Republic	Peru	Venezuela	Mexico	Puerto Rico	Total
N	2,937	2,009	1,933	1,961	2,002	2,002	12,844
Age (Mean, SD)	75.1 (7.0)	75.3 (7.5)	74.8 (7.4)	72.5 (6.9)	74.3 (6.7)	76.3 (7.4)	74.7 (7.2)
Women (N, %)	1,909 (65.0)	1,325 (66.0)	1,183 (61.2)	1,249 (63.7)	1,267 (63.3)	1,347 (67.3)	8,280 (64.5)
Education (N, %)							
None	75 (2.6)	392 (19.7)	121 (6.3)	155 (8.1)	554 (27.7)	72 (3.6)	1,369 (10.7)
Some	651 (22.2)	1,021 (51.3)	231 (12.1)	444 (23.1)	863 (43.2)	389 (19.5)	3,599 (28.2)
Primary	977 (33.4)	370 (18.6)	727 (37.9)	964 (50.2)	351 (17.6)	415 (20.8)	3,804 (29.8)
Secondary	728 (24.9)	135 (6.8)	517 (27.0)	266 (13.8)	124 (6.2)	713 (35.7)	2,483 (19.5)
Tertiary	498 (17.0)	73 (3.7)	321 (16.7)	93 (4.8)	108 (5.4)	410 (20.5)	1,503 (11.8)
Missing	8	18	16	39	2	3	86
Dementia (N, %)	322 (11.0)	242 (12.1)	166 (8.6)	142 (7.2)	179 (8.9)	233 (11.7)	1,284 (10.0)
Missing	0	0	0	0	0	9	9
Physical impairment (N, %)							
None	1,286 (43.9)	599 (29.8)	887 (45.9)	748 (38.8)	835 (41.7)	708 (35.4)	5,063 (39.6)
1–2	1,353 (46.2)	945 (47.1)	780 (40.4)	693 (35.9)	824 (41.2)	865 (43.2)	5,460 (42.7)
3+	292 (10.0)	464 (23.1)	264 (13.7)	488 (25.3)	343 (17.1)	429 (21.4)	2,280 (17.8)
Missing	6	1	2	32	0	0	41
Modified Fried phenotype frailty (N, %)	601 (20.5)	683 (34.0)	451 (23.3)	220 (11.2)	183 (9.1)	237 (11.8)	2,375 (18.5)
Multidimensional frailty (N, %)	976 (33.2)	942 (46.9)	524 (27.1)	405 (20.7)	592 (29.6)	447 (22.3)	3,886 (30.3)
Depression (N, %)	683 (23.3)	761 (37.9)	537 (27.8)	574 (29.3)	574 (28.7)	330 (16.5)	3,459 (26.9)

*Notes*: SD: standard deviation.

Table 2 reports the numbers and percentage of frailty at follow-up by depression status. Participants who had frailty at baseline were excluded. The percentages of both the modified Fried frailty phenotype and multidimensional frailty were higher in participants with depression at baseline than those without the condition across all countries.

**TABLE 2:** Depression Status at Baseline and Frailty at Follow-Upa

		Modified Fried Phenotype Frailty (2–4 Characteristics) at Follow-Up: N (%)		Multidimensional Frailty at Follow-Up: N (%)	
	Depression Status	No	Yes	No	Yes
Cuba	No depression	1,202 (87.7)	184 (13.3)	859 (71.1)	350 (29.0)
	Symptoms	231 (80.2)	57 (19.8)	182 (67.2)	89 (32.8)
Dominican Republic	No depression	454 (76.7)	138 (23.3)	326 (64.7)	178 (35.3)
	Symptoms	138 (60.5)	90 (39.5)	96 (52.5)	87 (47.5)
Peru	No depression	681 (84.1)	129 (15.9)	645 (84.8)	116 (15.2)
	Symptoms	166 (77.9)	47 (22.1)	174 (78.0)	49 (22.0)
Venezuela	No depression	804 (94.5)	47 (5.5)	663 (85.0)	117 (15.0)
	Symptoms	246 (86.9)	37 (13.1)	211 (83.7)	41 (16.3)
Mexico	No depression	787 (79.5)	203 (20.5)	523 (65.0)	282 (35.0)
	Symptoms	233 (65.6)	122 (34.4)	143 (55.2)	116 (44.8)
Puerto Rico	No depression	821 (82.8)	171 (17.2)	624 (68.9)	282 (31.1)
	Symptoms	87 (58.4)	62 (41.6)	98 (60.9)	63 (39.1)
**Across centers**	**No depression**	**4,749 (84.5)**	**872 (15.5)**	**3,640 (73.3)**	**1,325 (26.7)**
	**Symptoms**	**1,101 (72.6)**	**415 (27.4)**	**904 (67.0)**	**445 (33.0)**

*Notes*: ^a^Depression symptomatology is defined as having either EURO-D or ICD-10 depression.

The results of competing risk modeling are reported in Table 3. Depression was associated with an increased hazard of incident frailty. For the modified Fried phenotype defined by two or more characteristics, the unadjusted pooled estimate was 1.87 (95% confidence interval [CI]: 1.66, 2.10; Z-test = 10.56; p < 0.001), which was reduced to 1.59 (95% CI: 1.40, 1.80; Z-test = 7.29; p < 0.001) after taking into account sociodemographic factors, physical impairment, and dementia. The association was also found in the modified Fried phenotype defined by three or four characteristics (1.71; 95% CI: 1.24, 2.38; Z-test = 3.24; p = 0.001). For multidimensional frailty, the unadjusted effect size was 1.29 (95% CI: 1.16, 1.43; Z-test = 4.67; p < 0.001) and became 1.19 (95% CI: 1.06, 1.33; Z-test = 3.02; p = 0.002) after adjustment. Variations in hazard ratios across countries were also smaller when using the multidimensional frailty (I^2^ = 0.0) compared to the modified Fried phenotype (I^2^ = 63.5). All countries apart from Cuba showed a 20%–30% higher hazard of incident multidimensional frailty in those who had depression at baseline.

**TABLE 3:** Subdistribution Hazard Ratio for Incident Frailty in Participants With Depressive Symptomatology (either EURO-D or ICD-10 criteria) at Baseline

	Modified Fried Phenotype Frailty (2–4 Characteristics)			Modified Fried Phenotype Frailty (3–4 Characteristics)			Multidimensional Frailty		
	Model 1 SHR (95% CI)a	Model 2 SHR (95% CI)a	Model 3 SHR (95% CI)a	Model 1 SHR (95% CI)a	Model 2 SHR (95% CI)a	Model 3 SHR (95% CI)a	Model 1 SHR (95% CI)a	Model 2 SHR (95% CI)a	Model 3 SHR (95% CI)a
Cuba	1.41 (1.05, 1.90)	1.28 (0.94, 1.74)	1.24 (0.91, 1.69)	2.59 (1.20, 5.60)	2.24 (0.98, 5.12)	2.17 (0.93, 5.03)	1.00 (0.79, 1.25)	0.97 (0.77, 1.23)	0.99 (0.79, 1.26)
DR	1.91 (1.47, 2.47)	1.70 (1.30, 2.23)	1.54 (1.15, 2.08)	2.95 (1.66, 5.23)	2.50 (1.39, 4.48)	1.92 (0.99, 3.71)	1.46 (1.14, 1.87)	1.34 (1.03, 1.73)	1.32 (1.00, 1.73)
Peru	1.31 (0.95, 1.82)	1.31 (0.93, 1.84)	1.22 (0.86, 1.73)	0.57 (0.17, 1.93)	0.57 (0.16, 1.98)	0.34 (0.08, 1.44)	1.39 (1.00, 1.94)	1.38 (0.97, 1.96)	1.33 (0.93, 1.89)
Venezuela	3.00 (1.96, 4.60)	2.93 (1.89, 4.52)	2.65 (1.67, 4.22)	2.96 (0.75, 11.21)	2.35 (0.61, 9.02)	2.00 (0.43, 9.21)	1.29 (0.90, 1.85)	1.26 (0.87, 1.85)	1.23 (0.83, 1.82)
Mexico	1.72 (1.38, 2.15)	1.62 (1.29, 2.04)	1.52 (1.20, 1.93)	1.66 (0.90, 3.07)	1.59 (0.83, 3.08)	1.47 (0.79, 2.74)	1.36 (1.10, 1.69)	1.27 (1.02, 1.59)	1.23 (0.98, 1.54)
Puerto Rico	2.78 (2.11, 3.67)	2.52 (1.90, 3.35)	2.18 (1.61, 2.95)	3.38 (1.81, 6.31)	2.94 (1.52, 5.68)	2.18 (1.11, 4.26)	1.36 (1.04, 1.76)	1.32 (1.01, 1.74)	1.21 (0.91, 1.61)
**Pooled**	**1.87 (1.66, 2.10)**	**1.74 (1.54, 1.96)**	**1.59 (1.40, 1.80)**	**2.36 (1.75, 3.18)**	**2.09 (1.53, 2.86)**	**1.71 (1.24, 2.38)**	**1.29 (1.16, 1.43)**	**1.23 (1.10, 1.37)**	**1.19 (1.06, 1.33)**
**I****^2^**	**76.3**	**73.5**	**63.3**	**41.2**	**20.2**	**17.0**	**20.3**	**2.5**	**0.0**

*Notes*: Model 1: unadjusted; Model 2: adjusted for age, sex, and education level; Model 3: adjusted for age, sex, education level, number of physical impairments, and dementia. DR: Dominican Republic; SHR: subdistribution hazard ratio. ^a^Z-test for coefficients estimated in competing risk models and pooled estimates in meta-analysis.

The results of sensitivity analysis showed that the associations between depression and frailty were robust when excluding people with dementia at baseline (Supplementary Table S1). The effect sizes were generally similar to the main analysis.

## DISCUSSION

### Main Findings

This study investigated the potential impact of depression on incident frailty in older people from six Latin American countries and considered both modified Fried phenotype and multidimensional frailty. The results suggest that older people who had depression were more likely to develop frailty compared with those without depression. Depression, a highly prevalent condition in later life, was associated with a 60% increased hazard of modified Fried phenotype frailty and 20% for multidimensional frailty after adjusting for sociodemographic factors, physical impairments, and dementia and considering the competing outcome of mortality. This means that up to one-third of frailty could be attributed to depression in later life.

### Strengths and Limitations

Based on a population-based cohort study, this study included a large number of older people in six Latin American countries. Complete information on physical and mental health conditions were collected through standardized and structured interviews. The analysis included two types of frailty measures and used competing risk modeling to account for high mortality in later life.

This study had some potential limitations. Although the 10/66 sample was selected to be as representative as possible of the general population, it is based on catchment areas that are not nationally representative. This might affect generalizability of the results but the strong association between depression and frailty was clear across older people in different settings. A modified version of Fried phenotype frailty was used here^19^, ^20^ and the results might not be comparable to existing studies using the full Fried criteria. However, lack of grip strength information might not affect the results. Previous studies have reported that the association between grip strength and adverse health conditions was attenuated when adjusting for other frailty indicators and confounding factors.^28^, ^29^ Although the multidimensional criteria was used to incorporate a wide range of indicators related to frailty, some domains such as cognition and nutrition could be sensitive to cultural and environmental factors. Because of limited statistical power, the analysis did not test variation across countries, but 95% CIs largely overlapped. Some factors such as biomarkers for inflammation and dopamine could be mediators in the association between depression and frailty,^8^, ^11^ but were not investigated in this study owing to a lack of relevant measures in the 10/66 surveys.

### Interpretation of Results

This study suggests a negative impact of depression on incident frailty in later life and provides additional evidence from low- and middle-income countries. The negative relationship corresponds to a recent meta-analysis, which summarized eight cross-sectional studies and four longitudinal studies mainly based on older people living in high-income countries.^8^ Compared with the pooled estimates reported in the meta-analysis, the effect sizes found in this study were much smaller for both frailty definitions. Although a negative relationship between depression and frailty in later life has been consistently reported in study populations from different countries, it is noteworthy that the strength of associations could be related to variations in study designs and measurement methods.

The effect sizes were found to be different when using the modified Fried frailty phenotype and multidimensional criteria. This may have been driven by two explanations: 1) frailty measured using Fried criteria tries to capture a unidimensional latent trait summarized as a dichotomous syndrome. This definition has very strong theoretical and biological underpinnings, which may reflect an identifiable and shared bioinflammatory pathway between depression and frailty,^8^, ^11^ for example, C-reactive protein and interleukin-6 have been shown to be elevated in both people with frailty and people with depression;^30^, ^31^ and 2) one of the criteria of Fried frailty (exhaustion) is very common in people with depression.^32^ We therefore expected to see a stronger relationship between depression and this definition of frailty, compared with multidimensional frailty.

The results from this study suggest that depression in later life may increase the hazard of developing frailty. Depressive symptoms can cause changes in sleep, appetite, physical activity, reduction in help seeking behavior, and adherence to medical treatments.^33^, ^34^, ^35^ These psychological and behavior symptoms might lead to weakness, decreased energy, and accelerate declines in physiological systems such as immune system.^11^, ^36^, ^37^

## Implications and future research directions

Depression is a treatable condition and improving treatments for this common mental disorder in later life may be beneficial to reduction in disability and mortality^38^ as well as frailty prevention. Underlying mechanisms between depression and frailty need to be further explored to inform potential interventions. To clarify the causal relationship, longitudinal studies need to have frequent follow-up and robust measures for depression and frailty over a long time period. Future research may also consider the cumulative effect of depression through the life course. For example, long-term depression has been related to brain inflammation.^39^ Measurements for midlife depression may provide additional information to underpin potential pathways and clarify the role of inflammation in these two conditions.

## CONCLUSIONS

Depression appears to play a key role in the development of frailty in older adults living in Latin America. Underlying mechanisms between physical and mental health in later life need to be further investigated in future research.

## Appendix Supplementary materials

Image, application 1

## Footnotes

Supplementary material associated with this article can be found in the online version at https://doi.org/10.1016/j.jagp.2019.04.008.
