# Supplementary material for: Depression and Incidence of Frailty in Older People From Six Latin American Countries
Source: Am J Geriatr Psychiatry. 2019 Oct;27(10):1072–9. doi: 10.1016/j.jagp.2019.04.008 (PMC6742503; doi:10.1016/j.jagp.2019.04.008)
Supplement: Supplementary file 1 [file mmc1.docx]

**Depression and incidence of frailty in older people from six Latin American countries**

**Supporting Information**

**Table S1:** Numbers of Fried frailty characteristics across the six countries.

| Fried frailty | Cuba | Dominican Republic | Peru | Venezuela | Mexico | Puerto Rico | Total |
| --- | --- | --- | --- | --- | --- | --- | --- |
| 0 | 530 (31.7) | 198 (24.2) | 421 (41.2) | 725 (63.9) | 451 (33.5) | 408 (35.8) | 2733 (38.3) |
| 1 | 903 (53.9) | 394 (48.1) | 426 (41.6) | 325 (28.7) | 569 (42.3) | 500 (43.8) | 3117 (43.7) |
| 2 | 213 (12.7) | 182 (22.2) | 154 (15.1) | 75 0(6.6) | 282 (21.0) | 186 (16.3) | 1092 (15.3) |
| 3 | 25 0(1.5) | 44 0(5.4) | 19 0(1.9) | 8 0(0.7) | 42 0(3.1) | 43 0(3.8) | 181 0(2.5) |
| 4 | 3 0(0.2) | 2 0(0.2) | 3 0(0.3) | 1 0(0.1) | 1 0(0.1) | 4 0(0.4) | 14 0(0.2) |

**Table S2:** The relationship between depression and incident frailty in people without dementia.

|  | **Modified Fried phenotype frailty** | | | **Multi-dimensional frailty** | | |
| --- | --- | --- | --- | --- | --- | --- |
|  | **Model 1**  SHR  (95% CI) | **Model 2**  SHR  (95% CI) | **Model 3**  SHR  (95% CI) | **Model 1**  SHR  (95% CI) | **Model 2**  SHR  (95% CI) | **Model 3**  SHR  (95% CI) |
| Cuba | 1.56  (1.11, 2.19) | 1.47  (1.03, 2.09) | 1.43  (1.00, 2.04) | 1.04  (0.81, 1.34) | 1.13  (0.87, 1.47) | 1.15  (0.88, 1.50) |
| Dominican Republic | 1.90  (1.40, 2.57) | 1.73  (1.25, 2.40) | 1.55  (1.10, 2.20) | 1.48  (1.12, 1.97) | 1.36  (1.01, 1.83) | 1.29  (0.95, 1.76) |
| Peru | 1.34  (0.94, 1.92) | 1.32  (0.92, 1.90) | 1.28  (0.89, 1.85) | 1.38  (0.96, 2.00) | 1.39  (0.95, 2.04) | 1.31  (0.88, 1.94) |
| Venezuela | 2.91  (1.71, 4.94) | 3.04  (1.68, 5.49) | 2.91  (1.58, 5.38) | 1.16  (0.69, 1.95) | 1.43  (0.83, 2.45) | 1.33  (0.77, 2.32) |
| Mexico | 1.99  (1.54, 2.59) | 1.89  (1.45, 2.47) | 1.75  (1.23, 2.32) | 1.48  (1.16, 1.89) | 1.44  (1.11, 1.86) | 1.39  (1.07, 1.79) |
| Puerto Rico | 2.82  (2.07, 3.84) | 2.75  (2.00, 3.78) | 2.33  (1.66, 3.29) | 1.35  (1.00, 1.82) | 1.26  (0.92, 1.72) | 1.15  (0.83, 1.59) |
| **Pooled** | **1.97**  **(1.72, 2.25)** | **1.87**  **(1.63, 2.15)** | **1.71**  **(1.48, 1.98)** | **1.32**  **(1.17, 1.49)** | **1.31**  **(1.15, 1.49)** | **1.26**  **(1.11, 1.44)** |
| **I^2^** | **62.9** | **63.5** | **48.6** | **4.8** | **0.0** | **0.0** |

Competing risk model sub-hazard ratio (SHR) and 95% confidence interval (CI)

Model 1: unadjusted

Model 2: adjusted for age, gender and education level

Model 3: adjusted for age, gender, education level and number of physical impairments
